# American Health Primers

**Published:** 1879-11

**Authors:** 


### AMERICAN HEALH PRIMERS.

### EYE SIGHT AND HOW TO CARE FOR IT.

*By Geo. C. Harlan, M. D., Surgeon to the Will's Eye Hospital. Published by Lindsay & Blakiston.*

This is the fourth number of the primers. It treats upon a subject of great interest to every one who has eyes.

After the introduction, it treats upon the anatomy of the eye, the physiology of vision, the ophthalmoscope, injuries and diseases of the eye. Optical defects, spectacles, practical suggestions for the cure of the eye; effects of school life upon the sight.

This little work is compact, well and plainly written, free from technicalities; understandable by every one of even a moderate education. Every one can study it with interest and with profit.

The eye is a very important member of the body, and it should be guarded with most scrupulous care, and I know of no work better calculated to put one upon the right road, in this matter than this little work.

